# An antibody toolkit for the study of membrane traffic in *Drosophila melanogaster*

**DOI:** 10.1242/bio.018937

**Published:** 2016-06-02

**Authors:** Falko Riedel, Alison K. Gillingham, Cláudia Rosa-Ferreira, Antonio Galindo, Sean Munro

**Affiliations:** MRC Laboratory of Molecular Biology, Cambridge Biomedical Campus, Francis Crick Avenue, Cambridge CB2 0QH, UK

**Keywords:** Organelle markers, *Drosophila*, Golgi, Endosomes, Endoplasmic reticulum

## Abstract

The use of *Drosophila melanogaster* as a model organism has been pivotal to understanding the developmental processes of metazoans. However, the use of flies for studying subcellular organization is hampered by a paucity of reliable reagents to label specific organelles. Here, we describe the generation of mouse monoclonal antibodies against a set of markers of the secretory and endocytic pathways, along with goat polyclonal antibodies against two Golgi proteins. We show that the monoclonal antibodies are highly specific and sufficiently sensitive to detect endogenous proteins in crude extracts by immunoblotting with little background staining. By immunofluorescence the major compartments of the membrane traffic system (including the endoplasmic reticulum, the Golgi, and early and late endosomes) are labeled by at least one antibody. Moreover, the antibodies can be used to label organelles in fly tissues including salivary glands and wing imaginal discs. We anticipate that these antibodies will provide a useful tool kit to facilitate the investigation of how the endomembrane system functions and varies in the diverse tissue types of metazoans.

## Abstract (precis)

**Summary:** We report the generation and characterization of set of monoclonal and polyclonal antibodies for labeling the major compartments of the secretory and endocytic pathways in *Drosophila melanogaster*.

## INTRODUCTION

The model organism *Drosophila melanogaster* has proven invaluable for revealing the molecular basis of key developmental processes in metazoans with research aided by a cohort of genetic tools and reagents. In addition, *Drosophila* cultured cell lines have been successfully employed in numerous RNAi screens due to their high transfection efficiency and the ease of dsRNA production. There has been a long-standing interest in using *Drosophila* and their cultured cells to investigate the secretory and endocytic pathways. Cultured *Drosophila* cells have proven useful for investigating the fundamental principles of membrane traffic, especially as genome-wide RNAi screens are relatively tractable and not typically hampered by the potentially redundant paralogs that are often present in mammalian gene families (Kondylis and Rabouille, 2003; Kondylis et al., 2007; Wendler et al., 2010). Membrane traffic pathways have also proven important for many processes in development, including the release of key developmental signals, the down-regulation of signaling receptors, and the establishment of both apical-basal and planar polarity (Baron, 2012; Dunst et al., 2015; Fürthauer and González-Gaitán, 2009; Lu and Bilder, 2005; Zacharogianni and Rabouille, 2013; Zhang et al., 2007). Finally, there is a growing interest in understanding how the basic machinery of membrane traffic is varied and augmented in the diverse cell types of a whole animal (Burgess et al., 2011; Chan et al., 2011; Fox et al., 2010; Giansanti et al., 2007; Lerner et al., 2013). Indeed, many cells differ greatly in subcellular organization and secretory function, and the *Drosophila* system provides an excellent model to understand how this diversity is established.

Many proteins known to play important roles in membrane trafficking are conserved in *Drosophila* (Schlacht et al., 2014; Zhang et al., 2007). In addition, the general organization of the fly secretory and endocytic pathways is similar to that of a typical mammalian cell with the only notable difference being multiple discrete Golgi stacks scattered throughout the cytoplasm instead of a single ribbon-like Golgi, although the stacks maintain their cis*-*trans polarity (Kondylis and Rabouille, 2009). Thus, the combination of cell biology and genetics should make *Drosophila* ideal for understanding protein sorting and vesicle transport in the context of a whole organism. However, its use has been hampered by a paucity of widely available reagents to label intracellular organelles. *Drosophila* proteins have often diverged too far from their human orthologs to be recognized by mammalian reagents, and typically antibodies to *Drosophila* proteins are raised in rabbits which results in limited supply and restrictions on double labeling. The use of GFP-tagged proteins provides a valuable alternative approach but this requires genome engineering or, at the very least, genetic crosses with available stocks (Dunst et al., 2015).

We describe here a panel of monoclonal and polyclonal antibodies raised against proteins from the major organelles of the membrane trafficking pathways in flies to provide a useful and reliable set of reagents to label *Drosophila* organelles. These proteins are Calnexin99A [endoplasmic reticulum (ER)], Gmap (cis-Golgi), Golgin84 (Golgi rims), Golgin245 (trans-Golgi), Hrs (early endosomes), and Rab7 (late endosomes); and the antibodies are either mouse monoclonals or goat antisera to ensure supply and to enable double labeling with existing rabbit sera.

## RESULTS AND DISCUSSION

### Generation of antibodies against *Drosophila* target proteins

We identified proteins that have a well-defined localization in cells and are likely to be expressed in most cell types, thus making useful organelle markers. For the ER we chose calnexin (Cnx99A; CG11958), an integral membrane protein of the ER that acts as a chaperone in protein folding and quality control (Lloyd et al., 2002; Rosenbaum et al., 2006; Tan et al., 2009). For the Golgi we selected three coiled-coil proteins from different parts of the Golgi stack. In mammalian cells all three of these proteins are able to capture specific classes of transport vesicle and so appear to define destinations within the stack (Wong and Munro, 2014). Gmap (CG33206) is the *Drosophila* ortholog of mammalian GMAP-210 and is located to the cis-Golgi (Friggi-Grelin et al., 2006). Golgin84 (CG17785) is a coiled-coil protein with a C-terminal transmembrane domain that is located on the rims of Golgi stacks (Bascom et al., 1999). Golgin245 (CG3493) is the *Drosophila* ortholog of mammalian golgin-245 and is found on the trans-Golgi (Sinka et al., 2008).

For early endosomes we selected hepatocyte growth factor regulated tyrosine kinase substrate, Hrs (CG2903). Hrs is a component of the ESCRT-0 complex and is recruited to early endosomes by binding PtdIns(3)P where it contributes to the budding of intraluminal vesicles (Lloyd et al., 2002; Raiborg et al., 2001). For late endosomes we selected the small GTPase Rab7 (CG5915) (Sriram et al., 2003; Zhang et al., 2007). As for lysosomes, we have previously reported a rabbit antiserum against the GTPase Arl8 which effectively labels mature lysosomes in *Drosophila* cells and tissues (Hofmann and Munro, 2006; Wilkin et al., 2008).

To generate antigens we expressed all or part of the selected proteins in *E. coli* with hexa-histidine tags (Table 1). After purification under native or denaturing conditions the antigens were injected into mice or goats for the generation of either monoclonal or polyclonal antibodies, respectively. In the case of Rab7 a peptide was synthesized and this was used for immunization.

**Table 1.**
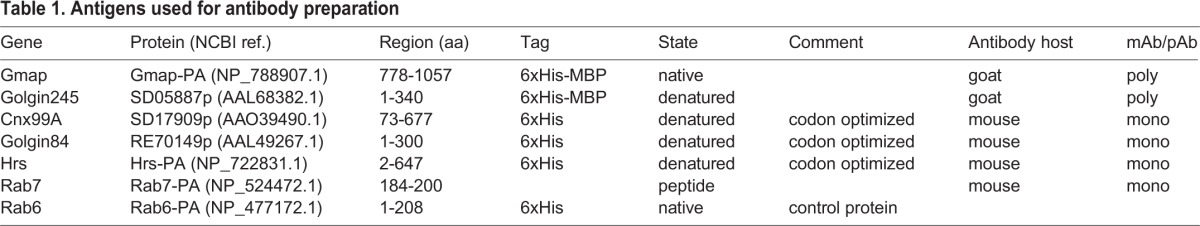
**Antigens used for antibody preparation**

### Antibody specificity and detection of endogenous proteins by immunoblotting

**Fig. 1.**
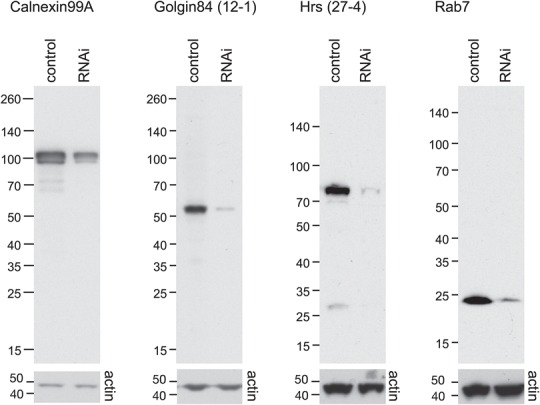
***Drosophila* antibodies are specific and can detect endogenous proteins by immunoblotting.** S2 lysates from cells treated with either control dsRNA or dsRNA against the target antigen were separated by SDS-PAGE and probed with the indicated antibodies (upper panels). Loading was assessed by blotting with an antibody to actin (lower panels).

The specificity of the mouse monoclonals or goat antisera was initially determined by immunoblotting *Drosophila* S2 cell lysates. Ten monoclonals corresponding to four antigens, or two goat antisera to each of two antigens were analyzed (Table 2). For the monoclonals, at least one clone against each antigen reacted primarily with a protein of the correct size in whole cell lysates (Fig. 1, Table 2). In each case this band was diminished when the cells were depleted of the antigen by RNA interference, confirming the specificity of the interaction (Fig. 1). Anti-Cnx99A is likely to cross-react with the closely related paralogs Cnx14D (72% identity) and CG1924 (73% identity), both of which have similar molecular weights. The sera raised against Gmap and Golgin245 gave several bands by immunoblotting and were therefore considered unsuitable for this application (data not shown).

**Table 2.**
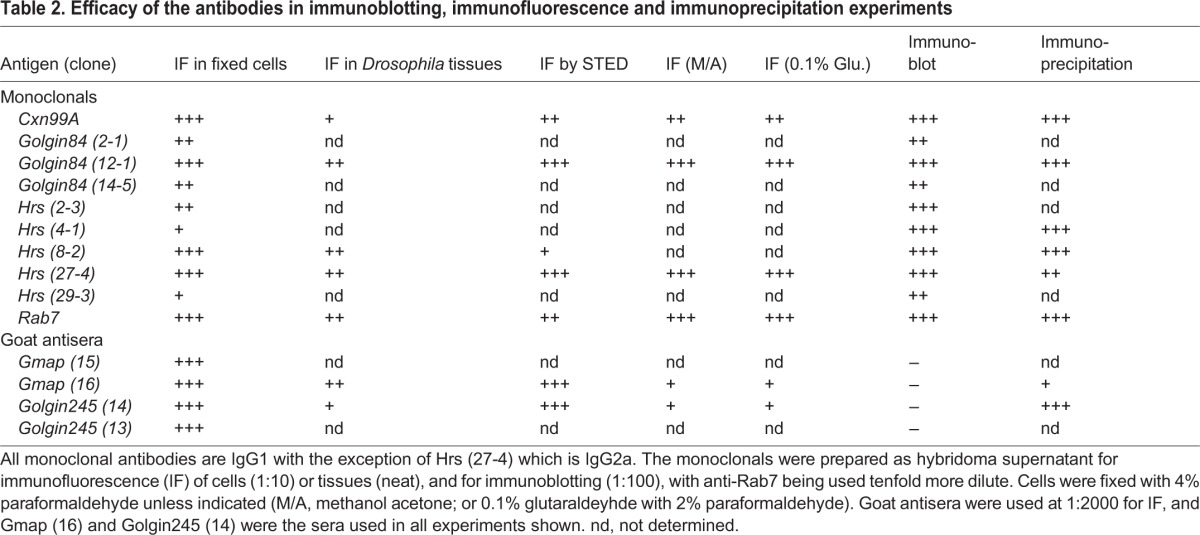
**Efficacy of the antibodies in immunoblotting, immunofluorescence and immunoprecipitation experiments**

### Immunofluorescence of secretory and endocytic organelles in *Drosophila* S2 cells

**Fig. 2.**
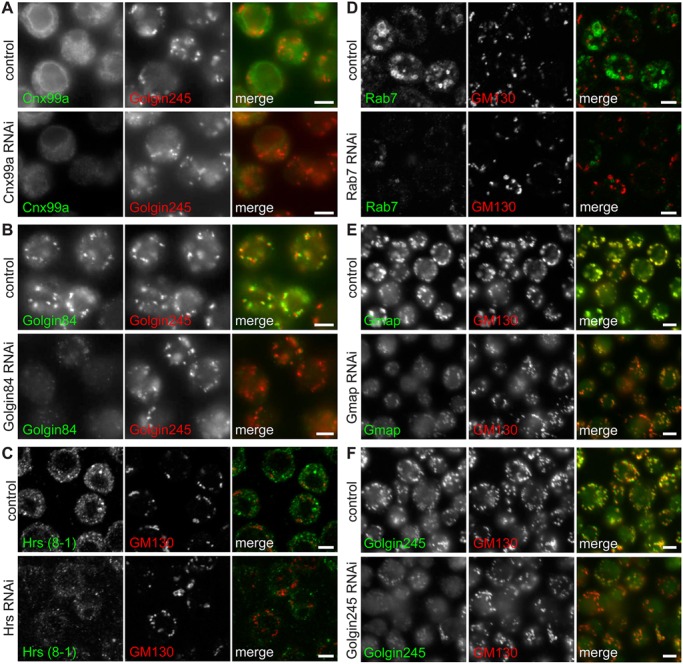
**Immunofluorescence of secretory and endocytic compartments in S2 cells.** S2 cells were fixed and stained with antibodies to Cnx99a (A), Golgin84 (B), Hrs (C), Rab7 (D), Gmap (E) and Golgin245 (F), along with rabbit antisera against the Golgi marker proteins Golgin245 or GM130 (red). Cells treated with dsRNA against the corresponding antigen had markedly reduced labeling with the specific antibodies whilst the Golgi marker staining was apparently unaffected (lower panels). Scale bars: 4 μm.

To test whether the antibodies were suitable for detecting native proteins in fixed cells, *Drosophila* S2 cells were fixed, permeabilized and stained with each antibody (Fig. 2). All gave labeling with the expected distribution, albeit with varying degrees of intensity. Labeling with the polyclonal antibodies against Gmap and Golgin245 was particularly bright with little background staining (Fig. 2E,F). In all cases this staining was greatly reduced following RNAi depletion of corresponding antigens, indicating that the antibody labeling is specific (Fig. 2, lower panels).

**Fig. 3.**
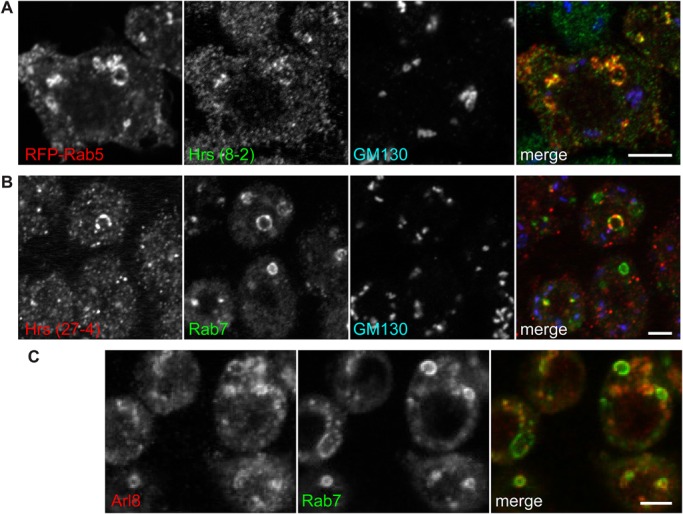
**Hrs and Rab7 labeling of endocytic compartments in S2 cells.** (A) Confocal micrographs of swollen endosomes induced by RFP-Rab5 overexpression in S2 cells, revealing labeling by antibodies against Hrs but not GM130. (B) Confocal micrographs of IgG-subclass-specific labeling of S2 cells with Hrs clone 27-4 (IgG2a) and Rab7 (IgG1), compared to the Golgi marker GM130. (C) S2 cells labeled with the monoclonal to Rab7 and an antiserum to the lysosomal marker Arl8. Scale bars: 4 μm.

Double labeling with previously reported antisera against Golgi markers confirmed that the monoclonals against Gmap, Golgin84 and Golgin245 all labeled the Golgi (Fig. 2B,E,F). To confirm that the antibodies to Hrs and Rab7 labeled endosomes, we compared their staining pattern to known markers. The monoclonal raised against Hrs labeled the enlarged early endosomes induced by overexpression of the GTPase Rab5 (Fig. 3A). Moreover, because the monoclonal to Hrs is a different IgG subclass to that against Rab7 we were able to compare their staining patterns. Labeling with Hrs-(27-4) (IgG2a) and Rab7 (IgG1) along with anti-GM130 antibodies demonstrated that each labeled a distinct membrane compartment (Fig. 3B). Moreover, Hrs-(27-4) and Rab7 labeled non-overlapping domains of a potentially ‘hybrid’ organelle that could be maturing from an early to late compartment. We also performed double labeling with the Rab7 monoclonal and a previously reported serum against the lysosomal marker Arl8 (Hofmann and Munro, 2006). Rab7 and Arl8 showed close association and partial overlap (Fig. 3C), consistent with the cycles of fusion and resolution between late endosomes and lysosomes, and also with a previous comparison of Rab7-GFP with Arl8 (Bright et al., 2005; Wilkin et al., 2008).

### Multi-color stimulated emission depletion (STED) microscopy in S2 cells

**Fig. 4.**
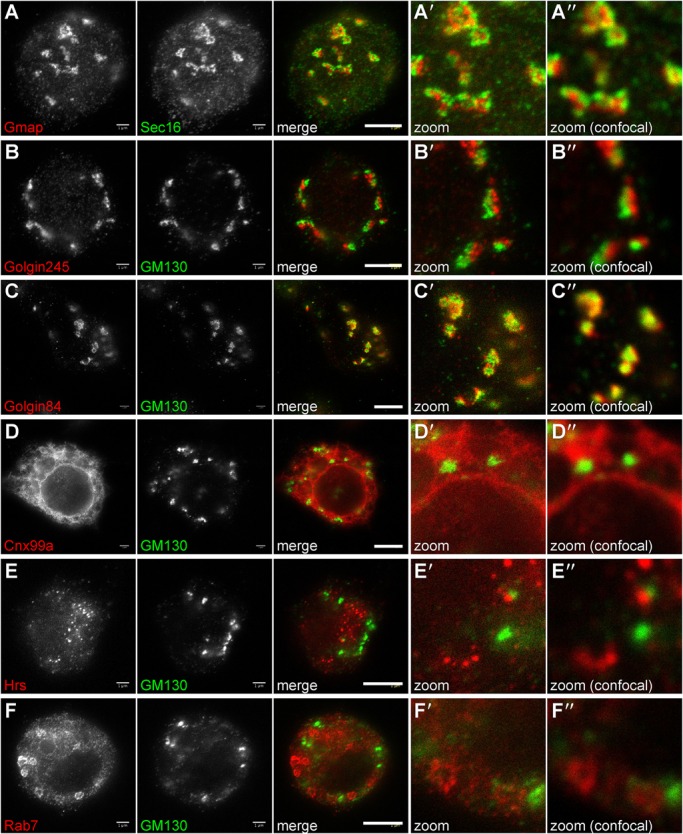
**Stimulated emission depletion (STED) microscopy in S2 cells.** Micrographs of S2 cells labeled with antibodies to Gmap (A), Golgin245 (B), Golgin84 (C), Cnx99a (D), Hrs (E) and Rab7 (F), imaged by standard confocal microscopy (Aʺ-Fʺ) or STED microscopy (A′-F′). The latter methods reveal fine details of each membrane compartment. Scale bars: 3 μm.

Stimulated emission depletion (STED) microscopy allows super resolution imaging by depleting fluorescence in specific regions, while leaving a focal spot active to emit fluorescence at the center. This allows imaging of biological samples at resolutions approaching 50 nm and has great potential to expand the information available from immunofluorescence imaging. However, due to the high light intensity required and the resulting photobleaching, the use of STED microscopy requires antibodies that give bright labeling with little background. To test the utility of the antibodies for STED microscopy, S2 cultured cells were stained with anti-GM130 or Sec16 to visualize the Golgi or ER exit sites respectively, and imaged using a Leica TCS SP8 STED microscope. Anti-Golgin84 clone 12-1, anti-Hrs clones 8-2 and 27-4, monoclonals to Cnx99A and Rab7 as well as antisera to Gmap and Golgin245 all provided sufficient sensitivity for STED microscopy and revealed finer details of their target organelles not visible by standard confocal microscopy (Fig. 4A-F).

### Labeling membranes in *Drosophila* tissues – salivary glands and wing imaginal discs

**Fig. 5.**
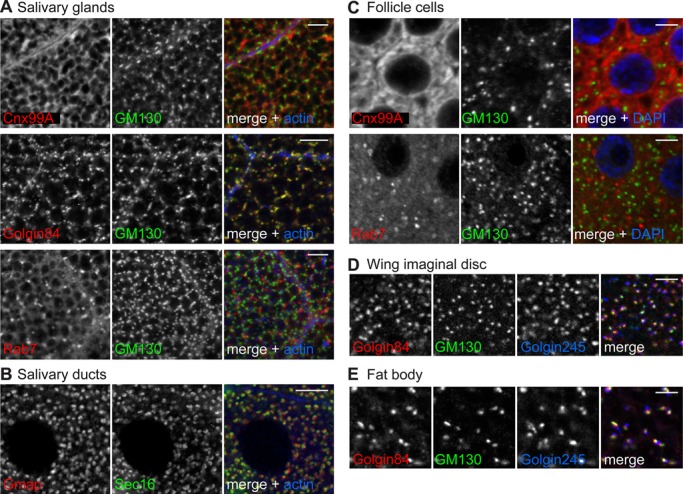
**Antibodies label organelles in *Drosophila* tissues.** Confocal micrographs of (A) salivary gland, (B) salivary duct, (C) ovarian follicle cells, (D) wing imaginal disc and (E) fat body, all double labeled with the indicated antibodies. Phalloidin or DAPI were used to label cortical actin or DNA to indicate cell boundaries or the nucleus, respectively. Scale bars: 10 μm (A,B) or 3 μm (C-E).

Organelle-specific antibodies that can label compartments in fly tissues are particularly useful as this allows the analysis of a diverse range of cell types, and offers the opportunity to examine the consequences of specific mutations. To test a range of tissues, salivary glands and ducts, the fat body, wing imaginal discs and ovaries were dissected from larvae, fixed, permeabilized and labeled with the antibodies reported here. The monoclonals against Cnx99A, Golgin84, Hrs and Rab7 all labeled discrete membranes in salivary glands and ducts (Fig. 5A and data not shown). Polyclonal antibodies against Gmap and Golgin245 also gave bright, distinct labeling of cis*-* and trans-Golgi compartments in these tissues (Fig. 5B and data not shown). Cnx99A and Rab7 produced the expected staining patterns in ovarian follicle cells (Fig. 5C), whilst the antibodies to Golgin245 and Golgin84 efficiently labeled the Golgi in both wing imaginal discs (Fig. 5D) and the fat body (Fig. 5E).

### Immunoprecipitation of GFP tagged antigens from whole cell lysates

**Fig. 6.**
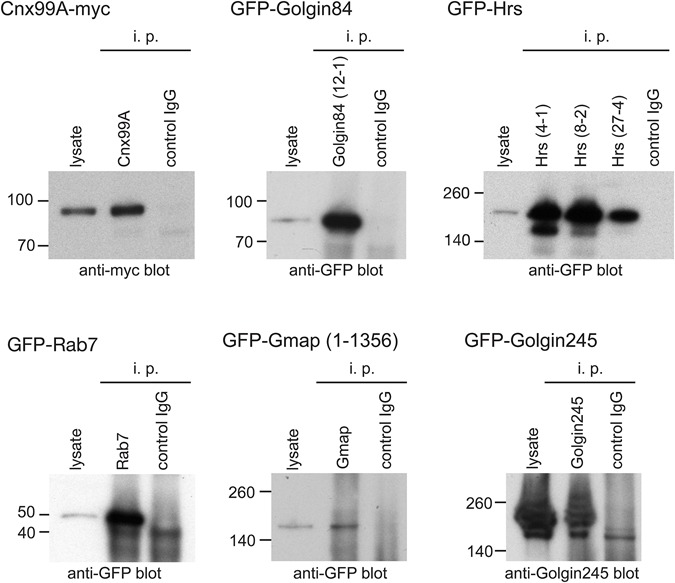
**Immunoprecipitation of tagged antigens from S2 cells.** Immunoblots of GFP- or myc-tagged proteins immunoprecipitated (i.p.) from lysates from transfected S2 cells using the indicated specific antibody and then probed for the relevant tag. In the case of Golgin245 the blot was probed with a previously described rabbit antiserum against the protein (Sinka et al., 2008). Lysates represent 7% of the input, and in each case control antibodies that should not bind were also tested.

Finally we tested whether the antibodies are suitable for immunoprecipitation of target proteins. To do this, lysates were prepared from S2 cells expressing tagged versions of either full-length or truncated Cnx99A, Hrs, Gmap, Rab7, Golgin84 or Golgin245. Each lysate was incubated with Protein G beads coupled to the relevant antibody or a control. Eluates were analyzed for the presence of tagged proteins by immunoblotting against the tag. Each bait was successfully isolated by its respective antibodies as indicated by a band of the right size that was absent from control lanes (Fig. 6, Table 2). Antibodies against Rab7, Hrs (clones 4-1 and 8-2) and Golgin84 were particularly effective, showing a strong enrichment of the tagged proteins over the starting lysate.

### Conclusion

This paper describes the generation and characterization of 14 antibodies against six marker proteins of the secretory and endocytic pathways in *Drosophila*. A summary of this data and the suitability of each antibody for the applications tested can be found in Table 2. In all cases we were able to show that the antibodies were specific for the endogenous protein using RNA interference and immunoblotting or immunofluorescence.

At least one clone for each antibody is suitable for staining a variety of *Drosophila* tissues including salivary glands and ducts, wing discs, fat bodies and follicle cells, and for STED microscopy (Table 2). Fortuitously one antibody against Hrs (27-4) is a different IgG subclass (IgG2a) compared with the others (IgG1) allowing double labeling with two monoclonals, or triple labeling with a suitable polyclonal antibody.

Collectively the antibodies should provide a useful resource for the study of many aspects of *Drosophila* cell biology including membrane trafficking and sorting, endocytosis, polarized cell growth and migration, cell signaling and development. These reagents will be made available to the scientific community through the non-profit Developmental Studies Hybridoma Bank (DSHB).

## MATERIALS AND METHODS

### Preparation of antigens

The antigens used to generate each antibody are shown in Table 1. Antigens for polyclonal antibodies were prepared as His_6_-MBP tagged proteins in the vector pETM-41 (Dümmler et al., 2005). Antigens for monoclonal antibodies were prepared as His_6_-tagged proteins in the expression vector pETM-11 except for Rab7 which was a peptide encompassing residues 184-200. The control used for removing monoclonal antibodies against the His_6_ tag alone was His_6_-Rab6 expressed from the vector pOPTH (gift from Olga Perisic, MRC LMB, Cambridge, UK). Fusion proteins were expressed in *E. coli* BL21-GOLD (DE3), or for His_6_-Rab6 in BL21-CodonPlus-RIL (both Agilent Technologies) and all were grown to an OD_600_ of 0.7 at 37°C before induction with 100-200 µM IPTG either overnight at 15°C or for 3 h at 37°C. His_6_-tagged antigens were prepared using the His Bind purification kit according to the manufacturer's instructions (Merck Millipore). Briefly, cells were harvested by centrifugation and lysed in binding buffer by dounce homogenisation and sonication. The lysates were clarified by centrifugation at 12,000×***g*** for 15 min and loaded onto Ni-NTA columns that were pre-equilibrated with binding buffer to isolate His_6_-tagged antigens. For Gmap purification, cells were pelleted and resuspended in binding buffer containing protease inhibitors and 20 mM imidazole. The column was washed with wash buffer containing 60 mM imidazole and eluted in 1 M imidazole. Hrs, Golgin245, Cnx99A and Golgin84 fusions were isolated as above except for the inclusion of 6-8 M urea in all buffers. The control protein His_6_-Rab6 was purified under native conditions using Ni-NTA beads (Qiagen) and eluted with 250 mM imidazole. Cnx99A, Golgin84, Golgin245, Gmap and Rab6 were desalted by gel filtration using PD-10 desalting columns (GE Healthcare) equilibrated with PBS. Hrs was dialyzed against a sequential gradient of PBS and urea, until all urea had been removed. In all cases, the purity and quantity of protein was assessed by SDS-PAGE and Coomassie blue staining with BSA standards.

### Generation of hybridomas and polyclonal sera

Purified antigens along with the control protein His_6_-Rab6 were provided to a commercial antibody company (BioGenes GMBH) at a concentration of 1 mg/ml and a purity of over 90% for generation of antibody-secreting hybridomas. Antibodies against Gmap and Golgin245 were raised in goats (two per antigen) by Cambridge Research Biochemicals.

### Cell culture, transfection and RNA interference

*Drosophila* S2 cells (D.Mel-2, Life Technologies) were grown at 25°C in serum-free medium (Express Five, Invitrogen) containing penicillin, streptomycin and L-glutamine, and regularly tested for contamination with mycoplasma. For expression of RFP-Rab5 S2 cells were transfected in 6-well plates with 1.5 µg plasmid and 1.5 µg carrier DNA [pAW empty vector (DGRC)] using Fugene HD (Promega) according to the manufacturer's instructions.

For RNA interference, *Drosophila* S2 cells were seeded in 6-well plates and transfected with 30 µg dsRNA against the target antigen using Transfast (Promega) according to the manufacturer's instructions. The region of the gene to target by dsRNA was determined using the Snapdragon RNAi design programme (http://www.flyrnai.org/snapdragon) and synthesized using the T7 Ribomax express RNAi system (Promega). Cells were incubated with dsRNA for 48-72 h prior to collection by cell scraping, washed once in PBS, pelleted and resuspended in SDS-PAGE sample buffer.

### Immunofluorescence of S2 cells by confocal microscopy and STED

For all images shown, cells were fixed for 20 min in 4% formaldehyde/PBS and permeabilized in 0.5% Triton X-100/PBS. For Table 2, cells were also fixed in methanol (5 min) and acetone (45 s), or as above but with 0.1% glutaraldehyde/2% paraformaldehyde/PBS and after permeabilization quenched with 1 mg/ml NaBH_4_ in PBS for 5 min. Cells were blocked for 30-60 min in PBS containing 20% FCS and 0.25% Tween-20 and probed with the antibodies in the same buffer. Rabbit antibodies against Arl8 (Hofmann and Munro, 2006), GM130 (Abcam, ab30637, 1:500 dilution), Golgin245 (Sinka et al., 2008), Sec16 (Ivan et al., 2008), and the mouse and goat (1:2000) antibodies generated in this study were detected with species-specific Alexa-labeled secondary antibodies (Molecular Probes, 1:400), or IgG subclass-specific secondary antibodies (Invitrogen). For standard imaging, cells were mounted in VECTASHIELD (Vector Laboratories) and micrographs obtained with a confocal microscope (Zeiss LSM 780) or a wide-field microscope with Micro-Manager software [Zeiss Axioplan and CoolSNAP HQ2 (Photometrics)]. For STED microscopy, coverslips were mounted in Prolong Gold anti-fade mounting media (Invitrogen). Super resolution STED images were acquired with a LEICA TCS SP8 X microscope equipped with 592 nm and 660 nm depletion lines.

### Immunofluorescence of salivary glands and imaginal wing discs

Third instar wandering larvae were collected in ice-cold PBS and the salivary glands, wing imaginal discs, ovaries and fat body dissected. All were fixed in 4% formaldehyde in PBS. Glands, fat body and ovaries were washed in PBS containing 0.3% Triton X-100 (PBST) before blocking in PBST plus 20% FBS followed by primary antibodies in the same buffer. Discs were washed in PBS with 0.05% (v/v) Triton X-100 and blocked in PBX250 [PBS, 0.05% (v/v) Triton X-100, 1 mg/ml BSA, 250 mM NaCl], with antibodies added in PBX buffer [PBS, 0.05% (v/v) Triton X-100, 1 mg/ml BSA]. All were incubated overnight at 4°C with agitation before washing and detection using species-specific Alexa-labeled secondary antibodies (Molecular Probes), and mounting in VECTASHIELD (Vector Laboratories) for confocal microscopy.

### Immunoprecipitation of GFP-tagged proteins

Hrs, Golgin84, Golgin245 (lacking the GRIP domain), Rab7 and Gmap (amino acids 1-1356) were inserted into a modified version of pAc5.1-V5-His (Invitrogen) containing an N-terminal GFP cassette and a stop codon before the V5 tag. Cnx99A was tagged with myc at the C-terminus using the Gateway^®^ vector pAWM (Life Technologies). S2 cells were plated in 75 cm^2^ flasks and transfected with 15 µg GFP-tagged construct and 15 µg carrier DNA using Fugene^HD^ (Roche) according to the manufacturer's instructions. Cells were incubated at 25°C for 48 h before scraping into ice-cold PBS, pelleting and resuspending in lysis buffer [20 mM Tris-HCl pH 7.4, 110 mM KCl, 1 mM EDTA, 1 mM DTT, 0.5% (w/v) CHAPS plus protease inhibitors]. Cells were incubated for 30 min at 4°C then clarified by centrifugation and pre-cleared by incubation with protein G PLUS-agarose (Santa Cruz) for 30 min at 4°C. Lysates were collected and incubated with 200 µl monoclonal tissue culture supernatant or 20 µl polyclonal goat serum for 2 h at 4°C with rotation. Protein G PLUS-agarose was added and the samples incubated for a further 2 h at 4°C with rotation. Beads were then washed in lysis buffer and isolated tagged proteins eluted with sample buffer. Samples were separated by SDS-PAGE, transferred to nitrocellulose and probed with mouse anti-GFP (Roche), rabbit anti-myc (Santa Cruz), or anti-Golgin245 antibodies.

### Deposition of reagents

The mouse monoclonals against Cnx99a, Rab7, Golgin84 (clone 12-1), and Hrs (clones 8-2 and 27-4), and the goat antisera against Gmap (16) and Golgin245 (14) have been deposited at the Developmental Studies Hybridoma Bank at the University of Iowa.
